# Supplementary figures and images for: CRISPR-based genome editing of a diurnal rodent, Nile grass rat (Arvicanthis niloticus)
Source: BMC Biol. 2024 Jul 2;22:144. doi: 10.1186/s12915-024-01943-9 (PMC11218167; doi:10.1186/s12915-024-01943-9)

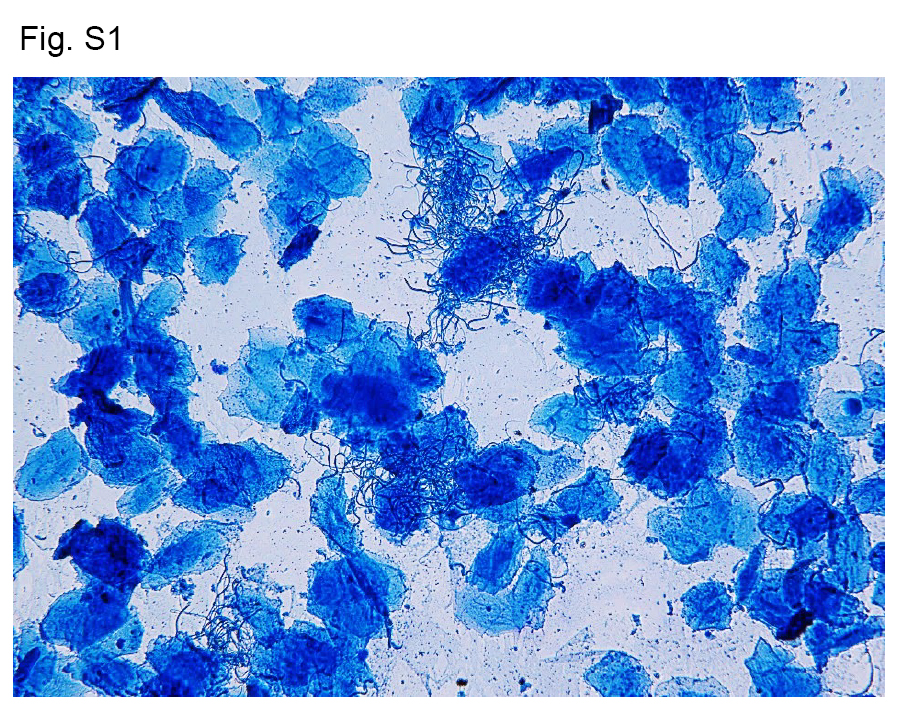

Supplement: Supplementary file 1 — Additional file 1: Fig. S1. Representative sperm-positive vaginal smear cytology of a female Nile grass rat. Sperm with a thin, elongated, hair-like appearance, and cornified epithelial cells, larger globular objects, are stained with methylene blue. [file 12915_2024_1943_MOESM1_ESM.jpg]

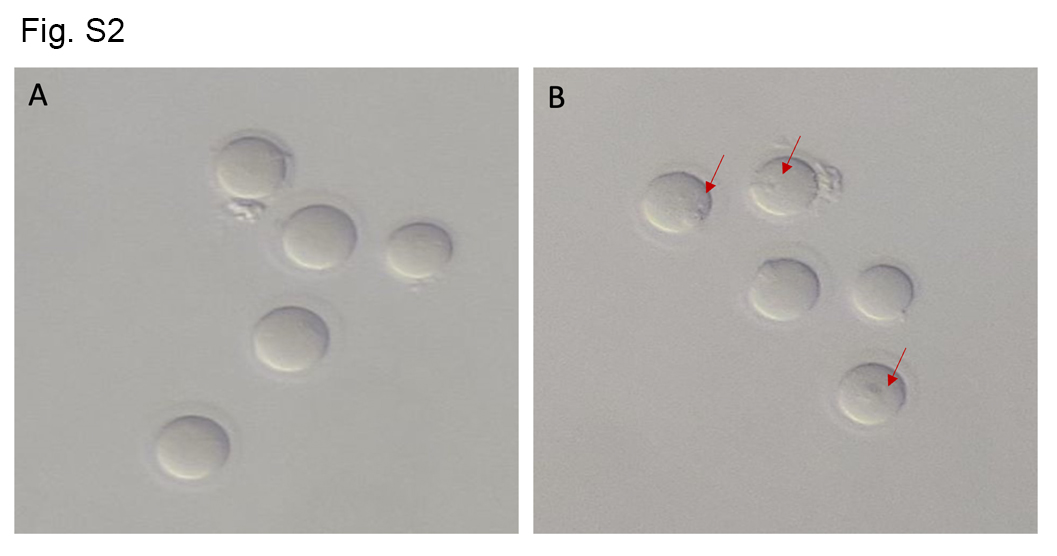

Supplement: Supplementary file 2 — Additional file 2: Fig. S2. Eggs retrieved from sperm-positive female grass rats the day after hCG administration and mating. A) No pronuclei are visible in eggs collected 19 h after hCG. B) Pronuclei (red arrows) are present in zygotes cultured to 27 h after hCG. [file 12915_2024_1943_MOESM2_ESM.jpg]

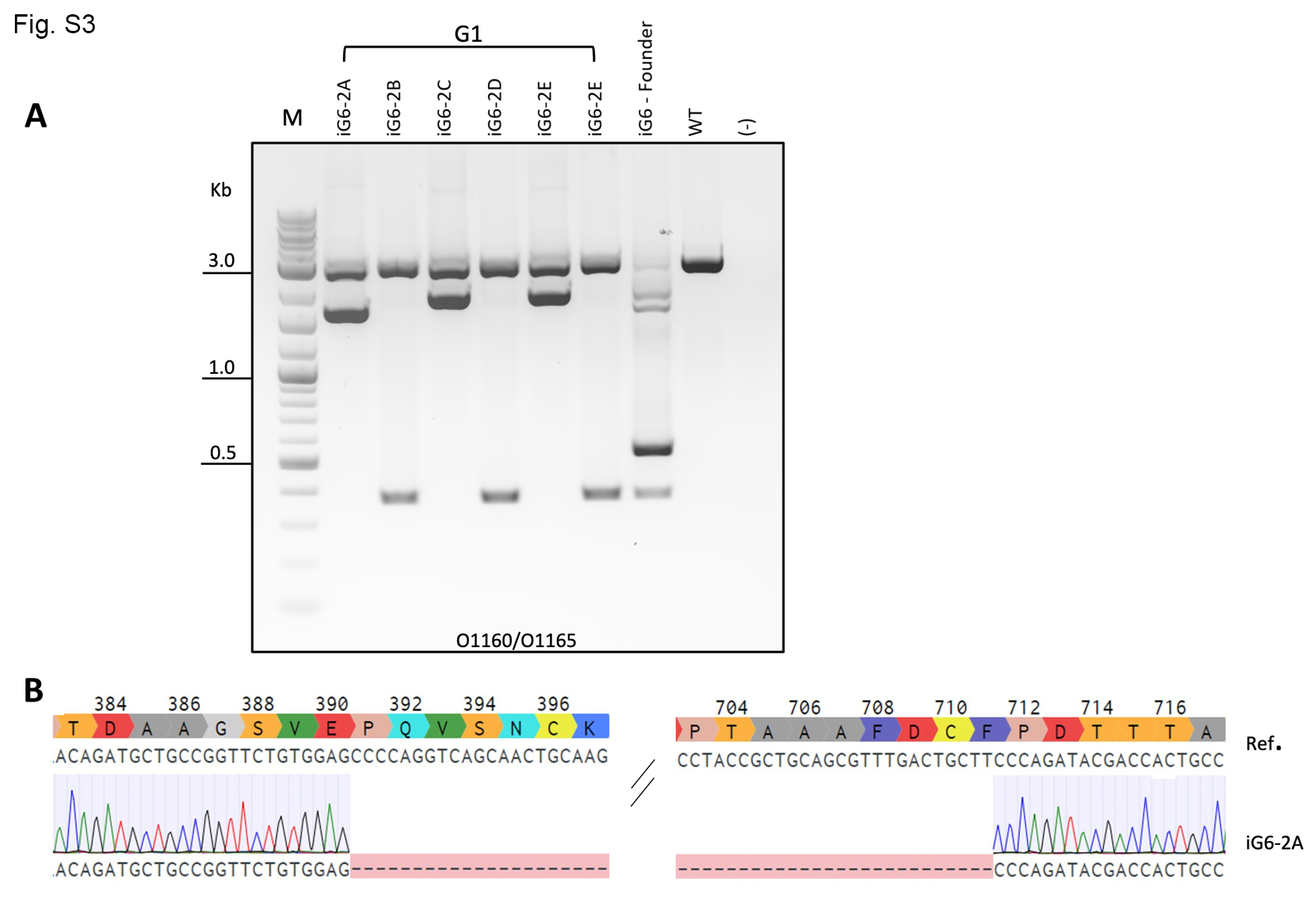

Supplement: Supplementary file 3 — Additional file 3: Fig. S3. Additional G1 transmission of Nile grass rat Rai1 deletion. A) Gel image showing long range PCR of G1 offspring from founder iG6 mated with a WT animal. Three different deletions were present in 6 pups assessed. B) Sanger sequence chromatogram of G1 animal iG6-2A shows a 962bp deletion, which results in frameshift after P391. [file 12915_2024_1943_MOESM3_ESM.jpg]
